# affyPara—a Bioconductor Package for Parallelized Preprocessing Algorithms of Affymetrix Microarray Data

**DOI:** 10.4137/bbi.s3060

**Published:** 2009-07-22

**Authors:** Markus Schmidberger, Esmeralda Vicedo, Ulrich Mansmann

**Affiliations:** Division of Biometrics and Bioinformatics, IBE, University of Munich, 81377 Munich, Germany. Email: markus.schmidberger@ibe.med.uni-muenchen.de

**Keywords:** parallel computing, R, microarray, preprocessing, normalization

## Abstract

Microarray data repositories as well as large clinical applications of gene expression allow to analyse several hundreds of microarrays at one time. The preprocessing of large amounts of microarrays is still a challenge. The algorithms are limited by the available computer hardware. For example, building classification or prognostic rules from large microarray sets will be very time consuming. Here, preprocessing has to be a part of the cross-validation and resampling strategy which is necessary to estimate the rule’s prediction quality honestly.

This paper proposes the new Bioconductor package **affyPara** for parallelized preprocessing of Affymetrix microarray data. Partition of data can be applied on arrays and parallelization of algorithms is a straightforward consequence. The partition of data and distribution to several nodes solves the main memory problems and accelerates preprocessing by up to the factor 20 for 200 or more arrays.

**affyPara** is a free and open source package, under GPL license, available form the Bioconductor project at www.bioconductor.org. A user guide and examples are provided with the package.

## Introduction

Studies of gene expression using high-density oligonucleotide microarrays have become standard in a variety of biological and clinical fields. They enable scientists to investigate the functional relationship between the cellular and physiological processes of organisms by studying transcription at genome-wide system levels. Affymetrix GeneChip® arrays are a very common variant of high-density oligonucleotide expression microarrays. The data recorded by means of the Affymetrix GeneChip® microarray technique are characterized by the typical levels of noise induced by the preparation, hybridization and measurement processes as well as a specific structure. Removing the sources of bias needs a specific preprocessing of the raw data as far as the steps background correction, normalization, and summarization are concerned. For more details and a brief introduction see e.g. Gentleman et al.^1^

The open source projects R^2^ and Bioconductor^3^ provide tools in computational biology and bioinformatics. R is a free software environment and provides a wide and extensible range of statistical and graphical techniques. Bioconductor is an open source software project and repository of instruments for the analysis and comprehension of genomic data. It is primarily based on the R programming language. Especially, for the preprocessing of microarrays the Bioconductor repository offers many tools which are implemented and stored in various packages^4^: **affy**^5^, **affyPLM**^6^, **vsn**^7^,... .

### Problems and challenges

Processing a large number of microarrays is generally limited by the available computer hardware. The main memory limits the number of arrays analysed. Furthermore, most of the existing preprocessing methods are very time consuming (http://bmbolstad.com/misc/ComputeRMAFAQ/size.html).^8^ Tasks which request a repeated preprocessing of large microarray sets may block computing devices for a long time. A specific class of such tasks is the building of classification or prognostic rules which uses resampling of the original data to estimate the classification or prognostic error.^9^

A further challenge is the fact that microarray experiments are becoming increasingly large. Meanwhile, large multi-centre studies — e.g. the Microarray Innovations in LEukaemia (MILE) study^10^ — prepare for a standardised introduction of gene expression profiling in diagnostic algorithms, aiming to translate this novel methodology into clinical routine for the benefit of patients with the complex disorders. In the MILE study, data of more than 2000 patients is used to derive diagnostic rules based on gene expression. To preprocess the data so called ‘Add-On normalization’ has to be used repeatedly.^11^ Add-On normalization allows to normalize a new microarray with respect to a normalization performed for a set off arrays. The **vsn** package^7^ offers this technique which is needed to apply gene expression profiles for classification or prognosis to a new patient. A misuse of this technique to normalize many of hundreds of patients to a base set of a few hundreds of patients results in a biased preprocessing result (see vignette of the **affyPara** package).

### Solutions

Most of these problems can be solved using faster computer processors and bigger main memories. Some preprocessing methods included in the **affy** and **affyPLM** packages try to solve these problems by exporting large parts of the algorithms to C routines. Alternatively one can buy business applications, store the data in databases, use the hard drive as main memory, or take advantage of the power of parallel computing. Algorithms using efficient data structures on the hard drive level already exist: **aroma.affymetrix**^12^. But, distributed computing is the most promising solution, because it accelerates the methods and it solves the main memory problems. Therefore, the **affyPara** package implements strategies using parallel computing for preprocessing of microarray data.

## Description

Parallel computing divides large computation problems into smaller ones, which are then solved concurrently. An overview, review and benchmark of parallel computing techniques and tools for parallel computing with R is available in Schmidberger et al.^13^

Parallel computing for microarray data distributes arrays to different processors, performs demanding computations on smaller sets of microarrays and communicates the results between the processors efficiently to achieve the needed overall result. Microarray data are stored in a matrix structure. Using the block cyclic distribution the arrays will be distributed equally to all nodes. Therefore, the amount of required main memory per processor gets smaller. Basic Bioconductor packages can be used on the single processors since their data structure is array oriented. The **snow** package^14^ is used as parallel computing API due to availability on different cluster environments, its compatibility to many multi-processor and multicomputer systems, its good performance, and user friendly code interface.^13^

**Figure 1.**
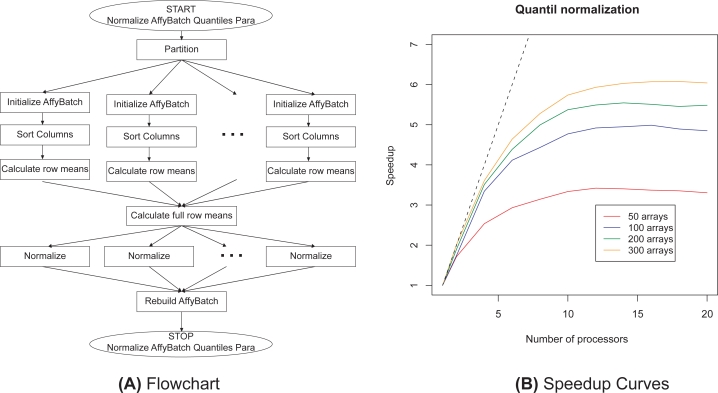
Flowchart and relative speedup curves for parallelized quantile normalization calculated on the super-computer HLRBII at the LRZ in Munich, Germany.

Existing statistical algorithms and data structures had to be adjusted and reformulated for parallel computing. Using the parallel infrastructure the methods could be enhanced and new methods will become available. For background correction the methods *RMA* and *MAS 5.0* are implemented. These methods only depend on the actual array and can easily be parallelized. Normalization methods make measurements from different arrays comparable and multi-chip methods have proved to perform very well.^4^ The normalization methods *contrast, invariantset, quantile*, and *vsn* (in development) are parallelized. The parallelization of the quantile normalization is visualized by the flowchart in Figure 1 (a). It was also possible to parallelize several summarization methods (*avgdiff, liwong, mas, medianpolish*) as well as complete preprocessing strategies (e.g. *rma*). Furthermore quality assessment tools optimized for huge numbers of microarrays are implemented.

The user-interface of the **affyPara** package is very similar to the code structure of the **affy** package and therefore very easy for trained R and Bioconductor users. Some simple example codes for creating an AffyBatch object and rma background correction with the **affy** and **affyPara** packages is visualized in the following code lines:

> *library (affy)*
> *AB* <*- ReadAffy()*
> *AB_bgc* <*- bg.correct(AB, method* = *“rma”)*
> *library (affyPara)*
> *makeCluster (5, tpe* = *“MPI”)*
> *AB* <*- ReadAffy()*
> *AB_bgc* <*- bgCorrectPara(AB, method = “rma”)*
> *stopCluster()*

To use the power of parallel computing, the user needs only a working computer cluster and cluster start or administration programs (e.g. Sun Grid Engine). The R syntax is very similar and only two more lines for starting and stopping the cluster are required.

## Results

In parallel computing, speedup (S) refers to how much a parallel algorithm is faster than a corresponding sequential algorithm: *S_N_* = *T*_1_/*T_N_*. Where *N* is the number of processors, *T*_1_ the execution time of the sequential algorithm and *T_N_* the execution time of the parallel algorithm with *N* processors.^15^ Limits for the speedup are described in ‘Amdahl’s Law’.^16^ For example in theory using *N* processors can not achieve a speedup of more than factor *N*.

Figure 1(b) visualizes the relative speedup for quantile normalization. The plot compares the parallelized and new implemented code in the **affyPara** package running on one processor to the execution time on two to twenty processors. Due to the use of 15 computers for 300 microarrays an absolute speedup can be achieved up to factor 20 (serial computation time of the **affy** package: 1806 sec, parallel computation time: 75 sec). A substantial gain in computation time is essential for a successful application of resampling techniques to the validation of gene expression profiling rules.

More details about the implementation, speedup of other methods and usage can be found in Schmidberger and Mansmann (2008)^8^ or the vignette of the package.

## Conclusion

The **affyPara** package implements parallelized and efficient preprocessing methods for a huge number of high-density oligonucleotide microarrays in the R language. For all parallelized methods using 5 to 10 processors (or more) and more than 150 microarrays there is an obvious acceleration than with the code from the **affy** package. The user-interface is simple and extends the functionality of the **affy** package. Using the **snow** package the new package works on computer clusters as well as on multi-core architectures. It supports standard preprocessing steps and provides tools for quality assessment of huge numbers of microarray data. The **affyPara** package is a free and open source package — under GPL license — and available form the Bioconductor project at www.bioconductor.org. A user guide and examples are provided with the package.
